# Xenogeneic Testicular Cell Vaccination Induces Long-Term Anti-Cancer Immunity in Mice

**DOI:** 10.3390/cimb47060443

**Published:** 2025-06-10

**Authors:** Victor I. Seledtsov, Ayana B. Dorzhieva, Adas Darinskas, Alexei A. von Delwig, Elena A. Blinova, Galina V. Seledtsova

**Affiliations:** 1 Petrovsky National Research Centre of Surgery, 119991 Moscow, Russia; 2 Institute for Fundamental and Clinical Immunology, 630099 Novosibirsk, Russia; dorzhieva-ayana@yandex.ru (A.B.D.); blinovaelena@yandex.ru (E.A.B.); galina-seledtsova@yandex.ru (G.V.S.); 3 Innovita Research Company, 06118 Vilnius, Lithuania; darinskas.adas@gmail.com

**Keywords:** cancer vaccine, cancer/testis antigens, xenogeneic vaccination, cancer immunity, carcinoma, melanoma

## Abstract

Cancer/testis antigen (CTA) gene products are expressed in most malignant tumours, while under normal conditions their expression is primarily restricted to testicular cells. In this study, we investigated the prophylactic application of a xenogeneic (ram-derived) testicular cell (TC) vaccine for cancer prevention in an experimental animal model. C57BL/6 mice were immunised three times with either xenogeneic (ram) or syngeneic (mouse) formaldehyde-fixed spermatogenic tissue-derived cells. Following vaccination, mice were implanted with live B16 melanoma or LLC carcinoma cells. Tumour-bearing mice were subsequently assessed for survival and immunological parameters indicative of anti-cancer immunity. Xenogeneic vaccination with TCs induced cross-reactive immune responses to both B16 melanoma and LLC carcinoma antigens (Ags), as determined by an MTT ((3-(4,5-dimethylthiazol-2-yl)-2,5-diphenyltetrazolium bromide) assay. Prophylactic vaccination with xenogeneic TCs (xTCs), but not syngeneic TCs (sTCs), significantly improved survival rates, with 30% of vaccinated mice surviving after LLC carcinoma implantation. The induced immunity was long-lasting as mice implanted with LLC carcinoma cells 3–6 months post-vaccination exhibited prolonged survival. Furthermore, lymphoid cells from surviving vaccinated mice were capable of adoptively transferring anti-cancer immunity to naïve animals, significantly increasing their survival rates upon subsequent LLC carcinoma cell implantation. Vaccinated mice bearing LLC tumours exhibited a reduction in regulatory CD4⁺CD25⁺Foxp3⁺ T cells in the spleen, with no effect observed in the central memory CD4⁺CD44⁺CD62L⁺ T-cell compartment. Moreover, vaccinated mice displayed increased interferon gamma (IFN-γ) levels in the blood, with no significant changes in interleukin-10 (IL-10) levels. Prophylactic vaccination with xenogeneic CTAs effectively induces long-term, stable anti-cancer immunity, demonstrating potential for future immunopreventive strategies.

## 1. Introduction

Tumour cells differ from normal cells in their surface antigenic composition, which can trigger immune responses capable of controlling tumour growth. However, immune tolerance and immunosuppressive mechanisms often allow tumours to evade destruction, necessitating novel immunotherapeutic strategies to enhance anti-tumour immunity [1].

Among the different classes of tumour-associated antigens, cancer/testis antigens (CTAs) occupy a unique niche. CTAs are normally only expressed in immune-privileged sites such as the testes and placenta but are aberrantly upregulated in various tumours, including liver, breast, pancreas, lung, and colorectal cancers [2,3,4]. The expression of CTAs in tumours is associated with oncogenic properties and tumour progression, making them promising targets for immunotherapy [5,6]. Importantly, CTAs are highly conserved across species, yet subtle structural differences between species make xenogeneic CTAs highly immunogenic, allowing them to overcome immune tolerance mechanisms that suppress responses to self-antigens [7]. Evidence suggests that xenogeneic vaccines can break immune tolerance by presenting antigens (Ags) as ‘altered self’, leading to robust T cell-mediated immunity against homologous tumour-associated Ags [8,9]. Earlier studies indicate that immunisation with xenogeneic tumour-associated Ags can generate cross-reactive immunity and suppress tumour growth in both preclinical and clinical settings [10,11]. This type of immunisation can disrupt immune tolerance, triggering CD4+ and CD8+ T-cell responses and significant antitumor activity [11]. However, most prior research has focused on mono-antigenic vaccines, which target only a single tumour Ag. Given the heterogeneity of tumour cell populations, monogenic or oligoantigenic vaccines may only eliminate subsets of tumour cells while allowing antigen-negative clones to escape immune attack, leading to disease progression [1]. Poly-antigenic vaccination strategies which simultaneously target multiple tumour Ags have been proposed as a superior approach to overcoming tumour antigenic heterogeneity [7]. However, an unmet need persists for safe, effective, and scalable poly-antigenic vaccine platforms capable of inducing long-term protective immunity against a wide spectrum of tumours.

This study aimed to investigate the potential of a xenogeneic corpuscular vaccine derived from ram spermatogenic tissue, which contains a full spectrum of CTAs, to induce long-term anti-cancer immunity. We hypothesise that this poly-antigenic vaccine will be capable of counteracting tumour development and progression by targeting a broad range of tumour cells expressing various CTAs. Ram testicular spermatogenic cells were chosen as a source of xenogeneic CTAs due to their high expression levels of a broad (full) spectrum of CTAs. Furthermore, the selection of ram tissue offers practical advantages, such as uniform age and the feasibility of standardised tissue collection processes. This makes it a convenient and scalable source for vaccine production.

In this study, we demonstrate that prophylactic immunisation with xenogeneic testicular cells is able to induce long-term, cross-protective anti-cancer immunity in mice. Using a murine model we evaluated survival outcomes, immune response parameters, and adoptive transfer experiments to determine the vaccine’s efficacy. Our findings represent a first step toward the development of a poly-antigenic CTA-based vaccine that may be applicable in the prevention of recurrent and heterogeneous cancers—a therapeutic niche that remains largely unfilled to date.

## 2. Materials and Methods

This research received approval from the Institutional Ethics Committee at the Institute of Fundamental and Clinical Immunology (Protocol No. 143 dated 29 November 2023).

### 2.1. Mice

Male and female C57BL/6 mice, aged 4 to 6 months and weighing 18–20 g, were used for all experiments. The animals were obtained from the breeding facility of the Goldberg Research Institute of Pharmacology and Regenerative Medicine (Tomsk, Russia). They were housed with access to autoclaved food and boiled water. All procedures involving animals were conducted in accordance with the legislation of the Russian Federation and Directive 2010/63/EU of the European Parliament and of the Council of 22 September 2010 on the protection of animals used for scientific purposes. Euthanasia was performed by cervical dislocation. In experiments involving the transfer of immune cells, donor and recipient animals of the same sex were used.

### 2.2. Tumour Cell Lines

The Lewis lung carcinoma (LLC, H-2^b^) and B16 melanoma (H-2^b^) cell lines were obtained from the N.N. Blokhin Cancer Centre (Moscow, Russia) and maintained in RPMI-1640 medium supplemented with 10% FCS, 2 mM L-glutamine, and antibiotics (all reagents were from Paneco, Moscow, Russia).

### 2.3. Preparation of Cellular Vaccines

Mature ram testicles were collected following castration of young animals weighing 15–20 kg and were immediately placed on ice. The spermatogenic tissue was then separated from the surrounding connective tissue sheaths using a surgical scalpel. The isolated tissue was cut into approximately 5 mm fragments using scissors. These tissue pieces were washed with cold distilled water to remove any residual blood. Cells were then gently extracted from the tissue fragments into pre-cooled phosphate-buffered saline (PBS) using a glass homogeniser. The resulting cell suspension was left to settle for 7–10 min to allow large aggregates to precipitate. The collected cells were transferred to a test tube and fixed with a 1% paraformaldehyde solution for 15 min at room temperature. Following fixation, the cells were washed three times in PBS by centrifugation. Cells from the spermatogenic tissue of mature mice, as well as from ram spleens, were prepared using the same method. All cell preparations intended for immunisation were stored frozen until use.

### 2.4. Immunisation of Mice and Tumour Implantation

Mice were immunised intramuscularly into the thigh with fixed xenogeneic or syngeneic testicular cells (TCs) or xenogeneic spleen cells (xSCs) at a dose of 5 × 10⁶ cells per mouse, administered three times at seven-day intervals. This immunisation protocol was considered effective in inducing adaptive immune responses, based on previously published data [8,9] as well as the results of our own specialised studies, which are not presented in this paper. Fourteen days after the final immunisation, the mice were subcutaneously implanted with LLC carcinoma or B16 melanoma cells in the anterior abdominal wall at a dose of 10^5^ cells per mouse. Survival was monitored daily from the day of tumour cell implantation.

### 2.5. Isolation of Cells from Lymphoid Organs and Cell-Mediated Immune Transfer

Cells were obtained from the spleen or lymph nodes by gently pressing the tissue into a cold medium using a glass homogeniser. The cell suspension was left to stand for 7–10 min to allow large aggregates to settle. After washing in cold PBS, the cells were counted and used in experiments. For cell-mediated immune transfer, cells isolated from vaccinated animals were administered intravenously into the orbital sinus of intact mice at a dose of 10⁷ cells per mouse.

### 2.6. Preparation of Antigenic Cell Lysates for Immunological Studies

Viable LLC and B16 cells were washed in cold PBS, counted, and stored at a concentration of 2 × 10⁶ cells/mL in a frozen state until use in experiments.

### 2.7. MTT Assay for Assessing Immunoreactivity

Spleen cells were cultured in a 96-well plate (2 × 10^5^ cells/well) in RPMI 1640 medium supplemented with 10% FCS, 2 mM L-glutamine, and antibiotics, in the presence of the thawed cells (1 × 10^5^ cells/well) as indicated below, in an atmosphere of 5% CO_2_ at 37.5 °C for 72 h. After this period, the plate was centrifuged at 1000 g for 5 min, the supernatant was replaced with fresh medium, and 50 μL of MTT (3-(4,5-dimethylthiazol-2-yl)-2,5-diphenyltetrazolium bromide) reagent (MTT assay kit, Abcam, Cambridge, UK) was added to each well. After 3 h of incubation, 150 μL of solvent (dimethyl sulfoxide, DMSO) was added to the precipitate. Colour development in the wells was recorded using a TriStar LB 941 plate reader (Berthold Technologies, Bad Wildbad, Germany) at a wavelength of 590 nm and expressed in optical density units. The intensity of the colouration was proportional to the number of viable cells in the wells.

### 2.8. Flow Cytometry

To determine the CD4^+^CD25^+^Foxp3^+^ and CD4^+^CD44^+^CD62L^+^ cell subpopulations, spleen cells were stained with fluorochrome-conjugated mouse monoclonal antibodies. The staining was performed using a combination of antibodies according to the protocol recommended by the manufacturer (BioLegend, San-Diego, CA, USA). The content of the cell populations was determined by flow cytometry using a BD FACSCalibur instrument.

### 2.9. Measurement of Interleukin 10 (IL-10) and Interferon Gamma (IFN-γ) Levels

The levels of IL-10 and IFNγ were measured in the plasma of mice using an enzyme-linked immunosorbent assay (ELISA) with test kits from Cloud-Clone Corp. (CCC, Wuhan, China), according to the manufacturer’s instructions.

### 2.10. Statistical Analysis

Each experimental and control group consisted of 10 mice. The presented data reflect the results of at least two identical experiments. Statistical analysis of the results was performed using GraphPad Prism 8 software (GraphPad Software, San Diego, CA, USA), applying the non-parametric Mann–Whitney test. Mouse survival analysis was conducted using the Kaplan–Meier method, and the significance of survival differences was assessed using the Mantel–Cox log-rank test.

## 3. Results

### 3.1. Vaccination with Xenogeneic Testicular Cells Induces Cross-Reactive Anti-Tumour Immunity

**Figure 1:**
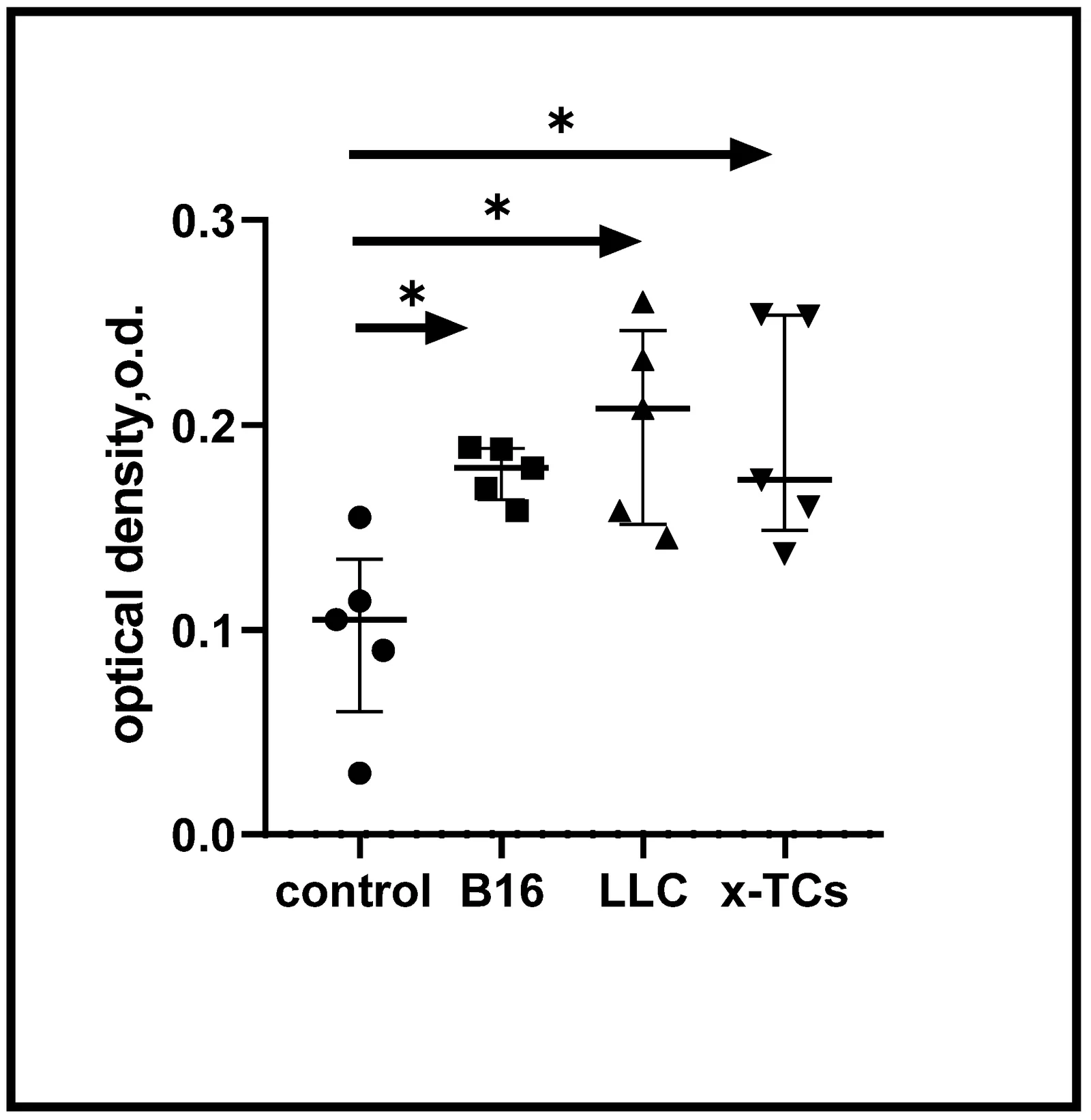
Immune responses of murine spleen cells to tumour-associated antigens. Spleen cells from xenogeneic TC-vaccinated mice, harvested 14 days post-final immunisation, underwent 72 h culture with or without (control) specified cell lysates (10^5^ cells/well). Cell viability was assessed using an MTT assay. * *p* < 0.05.

According to our initial hypothesis, immunisation of mice with xenogeneic CTAs should induce cross-reactive immune responses against syngeneic CTAs expressed on tumour cells. Figure 1 shows the enhanced immune (proliferative) reactivity of spleen cells from mice immunised three times with xenogeneic testicular cells (xTCs) in the presence of LLC carcinoma and B16 melanoma Ags. This immune reactivity was consistent and proportionate to the response of spleen cells to the vaccinal Ags. In control experiments, no immune reactivity was detected in spleen cells from unvaccinated animals). Overall, these data suggest the generation of cross-reactive anti-cancer immune responses in mice following immunisation with xTCs.

### 3.2. Prophylactic Vaccination with Xenogeneic Testicular Cells Enhances the Survival of Tumour-Bearing Mice

**Figure 2:**
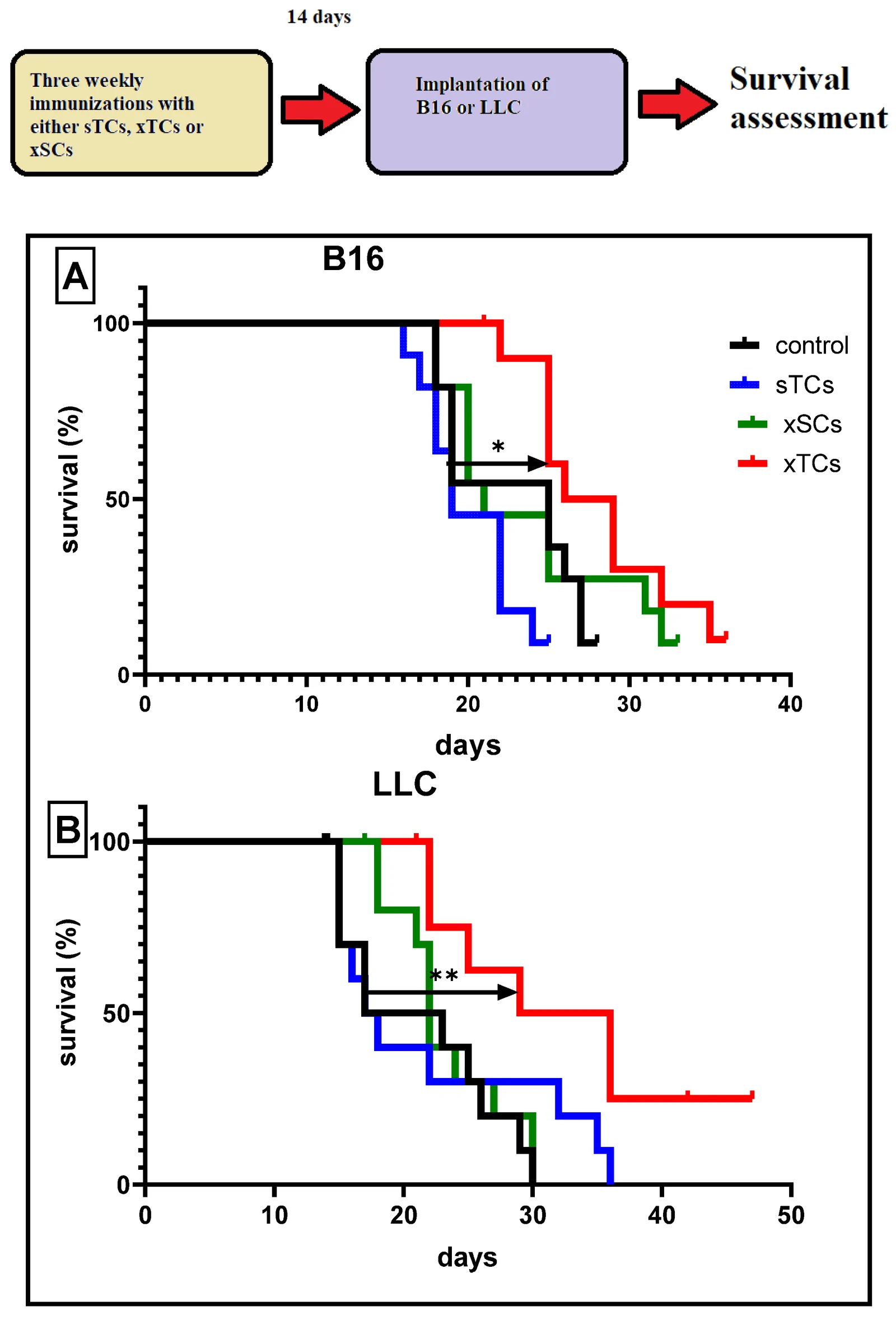
(**A**,**B**). Survival of mice with B16 melanoma (**A**) and LLC carcinoma (**B**). Mice were immunised three times with either xenogeneic testicular cells (xTCs), syngeneic testicular cells (sTCs), or xenogeneic spleen cells (xSCs) in a prophylactic regimen. Tumour cells (10^5^ cells/mouse) were implanted on day 14 following the final immunisation. The control group consisted of non-immunised animals. Mouse survival was analysed using the Kaplan–Meier method, and statistical significance was determined using the Mantel–Cox log-rank test. * n = 10, * *p* < 0.05, ** *p* < 0.01.

In subsequent experiments, we investigated whether xTC-induced cross-reactivity could impact tumour development. The data suggest that three immunisations with xTCs significantly increased the survival rates of mice implanted with B16 melanoma (Figure 2A) or LLC carcinoma (Figure 2B), as assessed 14 days after the final immunisation. Moreover, this protective effect was long-lasting, with 30% of vaccinated mice implanted with LLC carcinoma surviving throughout the entire observation period (6 months).

Importantly, control experiments showed that immunisation with syngeneic TCs (sTCs) or normal xenogeneic (ram) spleen cells (xSCs) did not affect survival rates. These findings are consistent with the interpretation that immune cross-reactivity induced by xenogeneic CTAs leads to the generation of significant anti-cancer immune reactivity.

**Figure 3:**
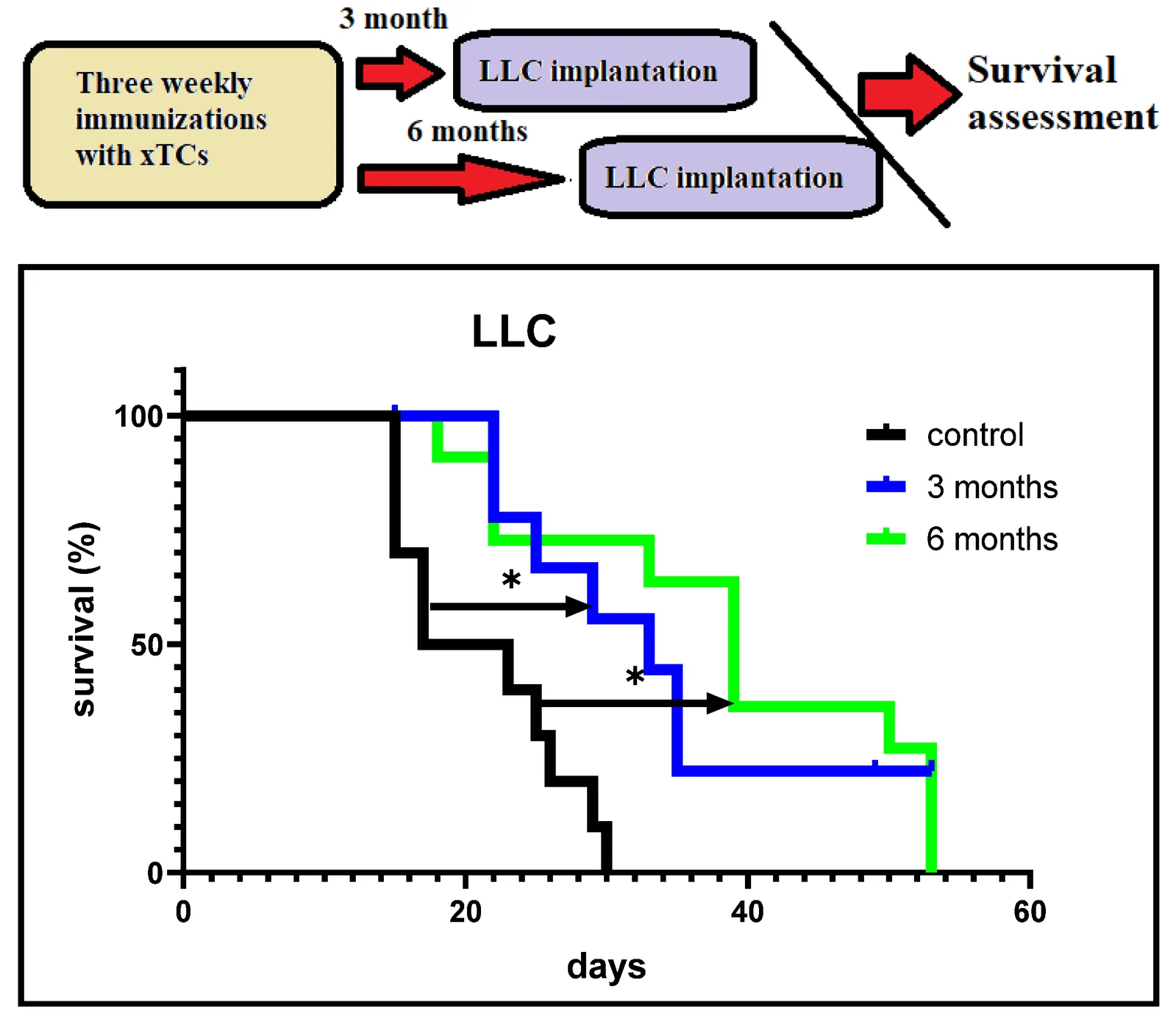
Survival of vaccinated mice depending on the timing of LLC carcinoma cell implantation. Mice vaccinated with xenogeneic TCs were implanted with LLC cells either 3 or 6 months after the final immunisation. The control group consisted of non-vaccinated mice. Survival was monitored following the implantation of LLC cells (10^5^ cells/mouse). Mouse survival was analysed using the Kaplan–Meier method, and statistical significance was assessed using the Mantel–Cox log-rank test. * n = 10, *p* < 0.05.

We also investigated the duration of the anti-cancer protection induced by xenogeneic CTAs. In these experiments, mice were implanted with LLC carcinoma cells 3 and 6 months after the last immunisation. Figure 3 shows significantly higher survival rates in both groups of vaccinated tumour-bearing mice compared to the control unvaccinated mice. Moreover, about 30% of vaccinated mice implanted with LLC carcinoma cells 3 months after the last vaccination survived, whereas no mice survived in the group implanted 6 months after the last immunisation. However, significantly higher survival rates were observed in the latter group compared to the control group.

Taken together, the data suggest that vaccination with xenogeneic CTA was highly effective in generating long-lasting, immune-mediated anti-cancer protection for up to 6 months.

**Figure 4:**
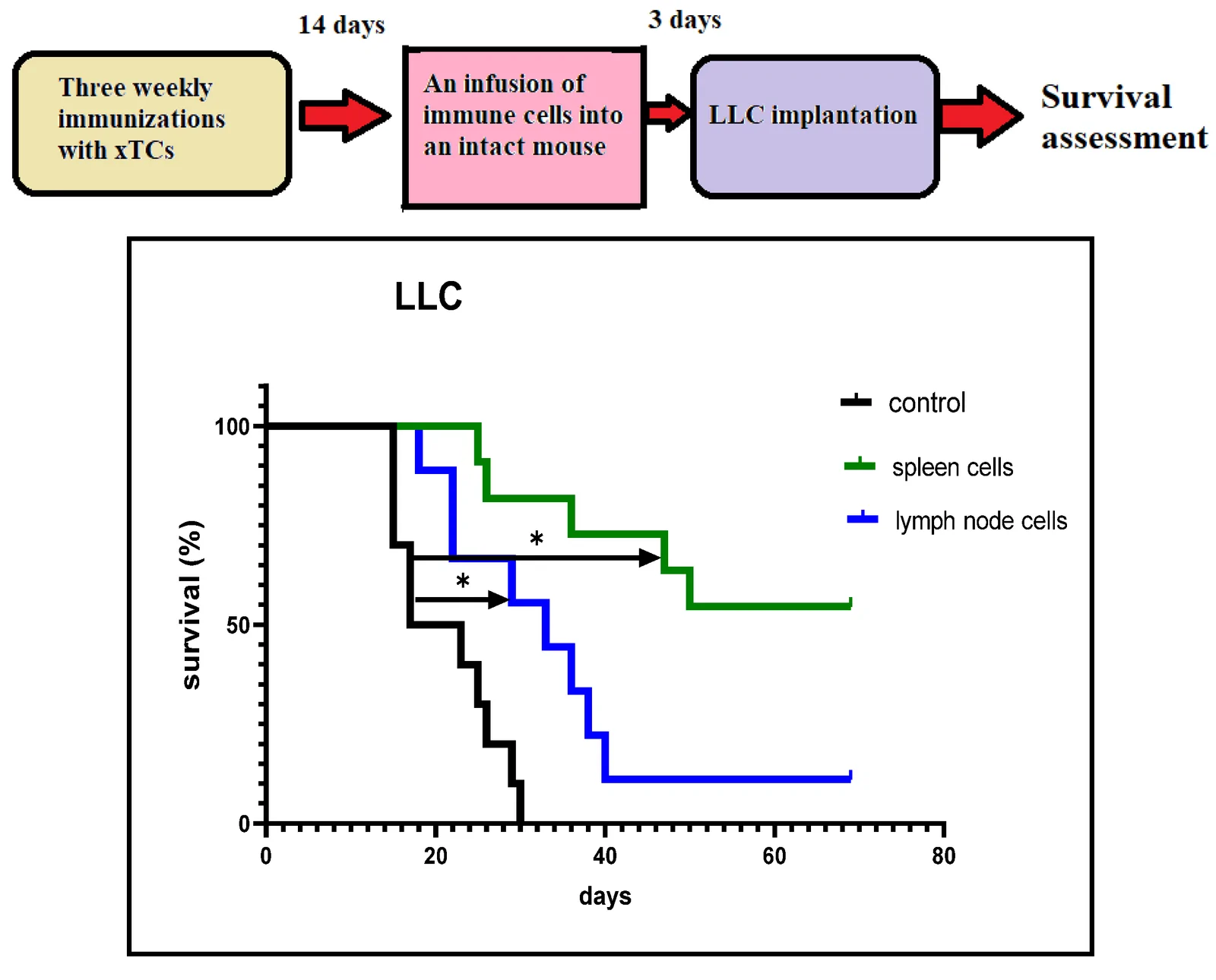
Survival curves from experiments involving adoptive transfer of anti-cancer immunity. Intact mice received intravenous injections of immune spleen or lymph node cells (10^7^ cells/mouse). Control mice did not receive immune cells. Survival was monitored following subcutaneous implantation of LLC carcinoma cells (10^5^ cells/mouse). Survival analysis was performed using the Kaplan–Meier method, and statistical significance was assessed with the Mantel–Cox log-rank test. n = 10, * *p* < 0.01.

We investigated the possibility of adoptive cell transfer of CTA-induced anti-cancer immunity. In these experiments, spleen or lymph node cells (10^7^/mouse) obtained from vaccinated mice 14 days after the last immunisation were injected intravenously into intact mice. Three days after the immune cell injection, LLC cells were implanted, and survival was assessed as described. Figure 4 shows significantly higher cancer survival rates in mice that received spleen/lymph node cells following implantation of LLC cells compared to the control mice. Furthermore, this effect was long-lasting, with approximately 20% of mice receiving lymph node cells and 50% of those receiving spleen cells remaining cancer-free by day 70 after implantation. These results led us to focus primarily on the immunological changes occurring in the spleen in response to vaccination, rather than in the lymph nodes.

### 3.3. Immunomodulatory Effects of Tumour Cells During Vaccination

**Figure 5:**
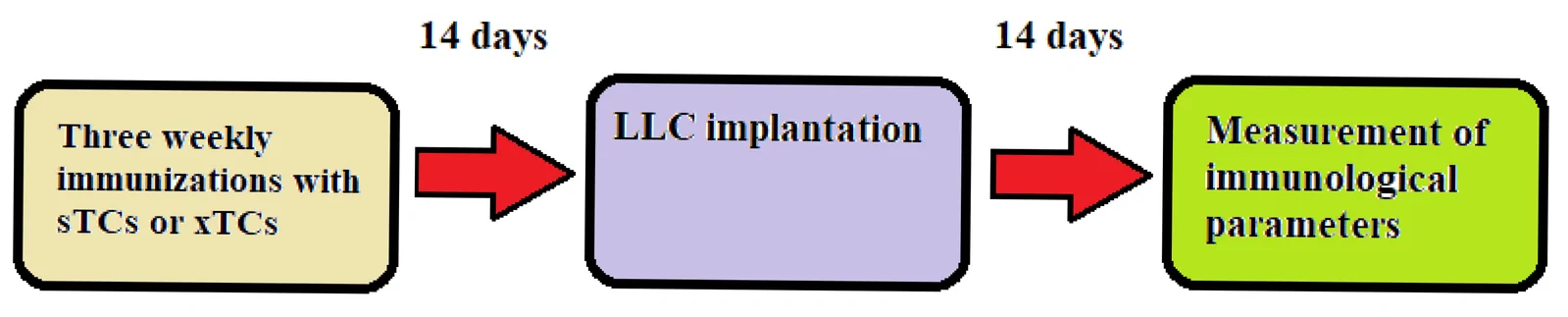
Scheme for the study of immunologic parameters in TC-immunised tumour-bearing mice.

It stands to reason that the primary application of a prophylactic systemic anti-cancer vaccination is to prevent the growth of cancer cells that have either already emerged or are likely to emerge as a result of cancerous mutagenesis. These cancer cells are capable of modulating the immune system in a way that prevents the development of tumour-specific adaptive immune reactivity. A similar situation could occur with tumour cells that remain in the body after a conditionally radical surgical removal of the primary tumour. Therefore, we further developed our experimental model to more closely reflect real-life scenarios in order to assess vaccine-induced changes in immunological parameters occurring in the presence of live tumour cells in the body. A schematic representation of this experimental model is shown in Figure 5.

Specifically, in these experiments we used mice immunised three times with sTCs or xTCs. Mice were implanted with LLC carcinoma cells (1 × 10^5^ cells/mouse) 14 days after the last immunisation. The content of regulatory T cells and memory T cells was measured in the spleen 14 days following implantation.

Flow cytometry data presented in Table 1 show that xenogeneic TCs vaccination reduced the percentage of regulatory CD4^+^CD25^+^FoxP3^+^ T cells in the spleen, with no effect on central memory CD4^+^CD44^+^CD62L^+^ T cells.

Additionally, the levels of IFN-γ and IL-10 were measured in the blood of TC-immunised mice. Our experiments revealed significantly higher IFN-γ levels in the sera of tumour-bearing mice immunised with xTCs compared to those observed in mice immunised with sTCs. No significant differences were observed in IL-10 levels between the two groups of mice (Table 2).

Taken together, the data obtained in this study suggest that vaccination with xenogeneic CTAs may create favourable conditions in the immune system for the development of anti-cancer immune reactivity.

## 4. Discussion

Surgical removal of the primary tumour remains the cornerstone of cancer treatment. However, this approach often carries an increased risk of disease recurrence. Similarly, certain age-related chronic pathological conditions are associated with a heightened susceptibility to tumorigenic diseases. At present, there are no effective systemic clinical or prophylactic strategies to mitigate the risk of tumorigenesis in patients who do not exhibit overt signs of disease. In this context, our study represents a foundational step towards developing a universal anti-cancer vaccine based on CTAs. A robust scientific framework supports this concept as over 200 different CTA proteins (MAGEA1, MAGE-A3, MAGE-A4, NY-ESO-1, PRAME, CT83, SSX2, etc.) have been identified to date. Notably, CTA-encoding genes play a critical role in organ and tissue formation during early ontogenesis. However, in later developmental stages these genes become inactive, with high CTA expression persisting only in spermatogenic testicular tissues and the placenta [3,4].

We have developed an anti-cancer vaccine prototype derived from spermatogenic ram cells, which contain the full spectrum of CTAs. These CTAs are highly xenogeneic relative to both mice and humans. Growing evidence indicates that xenogeneic vaccines can effectively overcome immune tolerance to weakly immunogenic homologous tumour-associated Ags [7,11]. Xenogeneic Ags can serve as an ‘altered self’, possessing enough dissimilarity from self-Ags to elicit an immune response while retaining sufficient similarity to ensure immune system recognition [11]. There is evidence that xenogeneic T-cell epitopes can bind host MHC molecules with greater affinity than native homologous epitopes, leading to the formation of more stable xenogeneic peptide/MHC complexes. This, in turn, enhances xenogeneic Ag-induced T-cell responses, which are cross-reactive with self-protein-derived Ags [11,12].

Cancer cells employ various mechanisms to evade immune detection, including the induction of immune tolerance and suppression. An important role is played by tumour-induced inflammation, which disables anti-tumour immunity and creates a favourable environment for tumour expansion [1,7]. In the context of xenogeneic vaccination, understanding these immune evasion strategies is crucial. Xenogeneic vaccines elicit anti-tumour immunity through multiple mechanisms, such as activating dendritic cells, promoting the maturation of antigen-presenting cells (APCs), and enhancing the differentiation of T cells into effector phenotypes. Additionally, these vaccines stimulate the production of pro-inflammatory cytokines such as IL-12, IFN-γ, and TNF-α, which collectively support cytotoxic T lymphocyte (CTL) responses and tumour cell elimination [13].

Although our xenogeneic vaccine was tested in an animal model, it was designed primarily for human application. A key consideration is that all humans possess natural (pre-existing) antibodies that mediate acute rejection of non-primate cells, serving as a major barrier to xenotransplantation. A significant proportion of these antibodies belong to the IgG class and specifically recognise the α-Gal epitope, which is abundantly expressed on glycoproteins and glycolipids of non-primate mammals and New World monkeys. These natural antibodies could enhance the immunogenicity of the vaccine by promoting the opsonisation and internalisation of xenogeneic vaccinal cells by APCs via Fcγ-receptor-mediated mechanisms. This process, in turn, facilitates cross-presentation of tumour-associated Ags to tumour-specific T lymphocytes [14].

It is well established that individual tumours are highly heterogeneous, consisting of antigenically diverse cancer cell subpopulations. Consequently, mono-antigenic or oligo-antigenic vaccination strategies may only target a subset of tumour cells while inadvertently selecting for Ag-negative variants with a growth advantage. We argue that only poly-antigenic immunisation can generate immune responses against a broad spectrum of tumour-associated Ags, thereby counteracting the intrinsic antigenic variability of tumours. This variability arises from the genetic instability of the tumour genome and its continuous interactions with the immune system. Vaccine-induced anti-cancer immunity would function optimally when the emergence of new tumour cell clones with altered antigenic profiles is counterbalanced by pre-existing polyclonal vaccine-induced immune reactivity. Consistent with this principle, our xenogeneic poly-antigenic vaccine contains the full repertoire of CTAs, promoting robust immune responses against cancer cell subpopulations expressing various CTAs.

Our vaccine falls into the category of cellular immunotherapeutic vaccines, engaging critical Ag processing and presentation pathways. The uptake of vaccine cells by APCs facilitates Ag cross-presentation to CD4+ and CD8+ T cells, thereby generating durable CD8+ T-cell memory with CD4+ T-cell help. Antigenic molecules presented on cell surfaces or within cellular compartments are generally more immunogenic than their soluble counterparts as the immune system is evolutionarily adapted to recognise and eliminate infected or malignant cells rather than harmless soluble Ags [15,16]. Published data also suggest that vaccinal cells can promote dendritic cell maturation, possibly due to the rapid degradation of cellular RNA and DNA into purine bases which are subsequently converted into uric acid. Uric acid, as a key endogenous danger signal, drives dendritic cell activation and enhances Ag presentation [17,18].

In our experiments, vaccination of mice with x-TCs induced effective anti-cancer immunity that suppressed tumour development for at least six months. Moreover, this immunity could be adoptively transferred to naïve animals using a relatively small number of immune cells from vaccinated mice. Most anti-cancer immune cells were localised in the spleen rather than lymph nodes, reinforcing the spleen’s pivotal role in regulating immune responses to tumour-associated Ags [19].

Prophylactic xTC vaccination lessened regulatory T cell prevalence, usually suppressing cytotoxic responses. This vaccine increased serum IFN-γ, a cytokine crucial for both adaptive and innate anti-cancer immunity [1]. These data indicate that vaccination with xenogeneic CTAs may favour different antitumor immune mechanisms.

Theoretically, breaking immune tolerance through poly-antigenic xenogeneic vaccination could increase the risk of autoimmune reactions. However, our vaccine is derived from spermatogenic ram tissue, which lacks tissue-specific Ags, reducing the likelihood of eliciting autoimmunity. Our observations confirm this assumption as vaccinated mice exhibited no signs of autoimmune disorders during the six-month observation period.

Normal spermatogenic tissue, as a source of vaccine antigens, offers a key advantage over tumour cell lines due to its genetic stability, maintained by natural mechanisms vital for species survival. This stability allows vaccine preparations derived from spermatogenic tissue to be standardised in accordance with the most stringent immunobiological requirements. Another important advantage of this tissue as a source of CTAs is the absence of mature tissue-specific Ags that could potentially trigger pathological, tissue-destructive autoimmune responses [20]. Importantly, ram-derived materials are broadly acceptable in many cultural and religious contexts, including Muslim-majority countries.

Despite its promise, our study has certain limitations. Notably, our xenogeneic cellular vaccine did not improve survival rates in tumour-bearing mice when administered therapeutically (i.e., after tumour cell implantation). This is likely due to the aggressive nature of the implanted B16 melanoma and LLC carcinoma, which rapidly led to host mortality before the immune response could fully develop. Prophylactic vaccination with xenogeneic CTAs elicited strong anti-cancer effects against LLC carcinoma and a statistically significant, albeit weaker, response against B16 melanoma. This discrepancy may be explained by the high intra-tumoral transcriptional heterogeneity of CTA expression in melanoma [21]. These findings suggest that the efficacy of xenogeneic CTA vaccine-induced immunity may vary depending on tumour type. However, various immunotherapeutic strategies—such as immunoadjuvants, cytokines, dendritic cells, and immune checkpoint inhibitors—could be employed to enhance the prophylactic and therapeutic potential of CTA-based vaccines against different cancers [22]. These approaches are currently under investigation, and the results will be reported in due course.

## 5. Conclusions

Xenogeneic (but not syngeneic) cancer/testis antigens can induce stable anti-cancer immunity in mice. Owing to the high levels of expression which are characteristic of cancer/testicular genes in most tumours, we envisage that a vaccine based on xenogeneic CTAs could potentially induce long-lasting anti-cancer immunity capable of counteracting the expansion of any tumour cells, regardless of their histogenesis. Therefore, a future adaptation of our lab’s cellular vaccine prototype could potentially be a universal anti-cancer prophylactic.

## Figures and Tables

**Table 1:** Percentage of regulatory CD4^+^CD25^+^FoxP3^+^ and central memory CD4^+^CD44^+^CD62L^+^ T cells in the spleens of TC-immunised tumour-bearing mice.

Cells	Immunisation with Syngeneic TCs	Immunisation with Xenogeneic TCs
CD4^+^CD25^+^FoxP3^+^	1.72 ± 0.35	1.2 ± 0.17 *
CD4^+^CD44^+^CD62L^+^	4.6 ± 2.4	4.8 ± 2.4

Notes: n = 6, * *p* < 0.01.

**Table 2:** Concentrations (pg/mL) of IFN-γ and IL-10 in the serum of TC-immunised tumour-bearing mice.

Cytokine	Immunisation with Syngeneic TCs	Immunisation with Xenogeneic TCs
IFN-γ	143.1 ± 24.62	247.4 ± 42.9 *
IL-10	13.42 ± 4.1	17.42 ± 1.7

Notes: n = 6, * *p* < 0.01.

## Data Availability

No special permission is required to reuse all or part of this article, including figures and tables.
