# Correction: Seledtsov et al. Xenogeneic Testicular Cell Vaccination Induces Long-Term Anti-Cancer Immunity in Mice. *Curr. Issues Mol. Biol.* 2025, *47*, 443

**DOI:** 10.3390/cimb48080790

**Published:** 2026-08-03

**Authors:** Victor I. Seledtsov, Ayana B. Dorzhieva, Adas Darinskas, Alexei A. von Delwig, Elena A. Blinova, Galina V. Seledtsova

**Affiliations:** 1 Petrovsky National Research Centre of Surgery, 119991 Moscow, Russia; 2 Institute for Fundamental and Clinical Immunology, 630099 Novosibirsk, Russia; dorzhieva-ayana@yandex.ru (A.B.D.); blinovaelena@yandex.ru (E.A.B.); galina-seledtsova@yandex.ru (G.V.S.); 3 Innovita Research Company, 06118 Vilnius, Lithuania; darinskas.adas@gmail.com

**Error in Affiliation**

The previous publication [1] contained an outdated (incorrect) affiliation for Adas Darinskas (third author). The proper affiliation is Innovita Research Company, 06118 Vilnius, Lithuania.

**Institutional Review Board Statement:**

The following information was included in the Institutional Review Board Statement: “This research received approval from the Institutional Ethics Committee at the Institute of Fundamental and Clinical Immunology (Protocol No. 143 dated 29 November 2023).”

**Conflicts of Interest:**

Author Adas Darinskas was employed by the company, Innovita Research Company. The remaining authors declare that the research was conducted in the absence of any commercial or financial relationships that could be construed as a potential conflict of interest.

The authors state that the scientific conclusions are unaffected. This correction was approved by the Academic Editor. The original publication has also been updated.
